# Evaluating Musculoskeletal Physiotherapist's Self‐Perceived Knowledge and Understanding of the Assessment, Diagnosis and Management of Degenerative Cervical Myelopathy: A Cross‐Sectional Survey

**DOI:** 10.1002/msc.70157

**Published:** 2025-08-03

**Authors:** Esther Dawson, Fi Macintosh

**Affiliations:** ^1^ York St John University York UK

**Keywords:** DCM, degenerative cervical myelopathy, musculoskeletal, physiotherapy

## Abstract

**Background:**

Degenerative cervical myelopathy (DCM) is often inadequately managed, leading to serious long‐term consequences for patients. Limited awareness of DCM among healthcare professionals may contribute to this problem.

**Objective:**

This study aimed to assess UK‐based musculoskeletal (MSK) physiotherapists' knowledge and confidence in the assessment, diagnosis, and management of DCM.

**Methods:**

An online cross‐sectional survey was conducted to gather data on MSK physiotherapists' understanding of DCM. It also explored perceived barriers to care and the value of additional training. The survey was distributed via the Chartered Society of Physiotherapy's (iCSP) website, professional networks, and social media. Responses were collected in March 2024.

**Results:**

A total of 108 physiotherapists participated, most with over 10 years of experience. Many reported that they had not received DCM‐specific training in their undergraduate programs. While most were fairly or very confident in their knowledge of DCM, subjective questioning relating to DCM, and management, nearly half expressed concern about missing a DCM diagnosis. Most participants felt they would benefit from further training.

**Conclusion:**

MSK physiotherapists are generally confident in their assessment and management of DCM but seek more comprehensive training, especially in symptomology and non‐surgical management. The findings of this survey also suggest a gap in DCM education at the undergraduate level, highlighting an opportunity to improve training for early‐career physiotherapists.

## 1 Introduction

Degenerative cervical myelopathy (DCM) arises due to age‐related degeneration of the cervical spine. These changes cause a narrowing of the space surrounding the spinal cord, leading to cord compression and subsequent injury (Myelopathy.org, 2023). Due to the spinal cord's limited regenerative capacity, recovery is minimal once damage occurs, often resulting in permanent symptoms (Hilton et al. 2019).

The current prevalence of DCM is estimated to be around 2% of the adult population or approximately 1 in 50 adults (Smith et al. 2021), with the rising prevalence linked to increasing population longevity (Fehlings et al. 2015). However, these figures are likely underestimated and influenced by factors such as the location of data collection (e.g., tertiary spinal clinics) and the high rates of underdiagnosis due to complex symptomatology and limited knowledge and awareness of early presentations (Davies, Mowforth, et al. 2022).

Treatment strategies for DCM, most commonly surgical, aim to prevent further neurological damage rather than reverse existing symptoms. Consequently, early diagnosis is associated with improved post‐operative outcomes, with one study identifying the duration of symptoms before surgical intervention as the only significant predictor of outcome (Ebersold et al. 1995). This finding is supported by additional research (Tetreault et al. 2015, 2019), with one study suggesting that the best outcomes are achieved when decompression surgery is performed within 6–12 months of symptom onset, while delays are linked to poorer long‐term results (Behrbalk et al. 2013). However, evidence points to chronic under‐management of DCM within the healthcare system (Davies, Mowforth, et al. 2022), with diagnostic delays averaging between two and 5 years, and in some cases extending up to 8 years (Behrbalk et al. 2013; Hilton et al. 2019; Pope et al. 2020).

The consequences of missed or delayed diagnosis of DCM are profound, both for patients and for society. Long‐term symptoms can vary from mild limb weakness to complete paralysis (Myelopathy.org, 2023), leading to a significant reduction in patients' quality of life (King et al. 2003). One survey found that DCM impacts quality of life more severely than conditions such as diabetes or cancer (Oh et al. 2017). Moreover, DCM affects not only the patient but also has a wider impact on the quality of life of their families (Mowforth et al. 2019). A recent report from the UK provided the first estimate of the annual cost of DCM, amounting to £685 million (Davies, Phillips, et al. 2022).

The international initiative, 'REsearch objectives and COmmon Data Elements for Degenerative Cervical Myelopathy’ (RECODE‐DCM), has identified the need for greater awareness of DCM as one of its top 10 priorities (AO Spine, 2023). Musculoskeletal (MSK) physiotherapists frequently encounter potential DCM patients and are well‐placed to contribute to improvements in the DCM care pathway. However, there is a notable lack of research regarding their awareness of DCM.

This study explored the current levels of awareness, understanding, and perceived barriers to the assessment, diagnosis, and management of DCM within musculoskeletal (MSK) physiotherapy as well as the need for additional training.

## 2 Objectives

To establish:

1. MSK physiotherapists' current levels of knowledge and awareness regarding assessment, diagnosis, and management of DCM.
2. Self‐perceived educational and training needs of MSK physiotherapists for assessing, diagnosing, and managing DCM.
3. Self‐perceived barriers in providing assessment, diagnosis, and management of DCM.

## 3 Methodology

A broad overview was required, so a quantitative approach was thought most appropriate to meet the study's aims. An online anonymous cross‐sectional questionnaire survey was developed and reported using the Checklist for Reporting Results of Internet E surveys (CHERRIES) (Eysenbach 2004).

### 3.1 Survey Development & Validity Testing

The survey was developed using Qualtrics software. The author designed and refined the questions based on a literature review. The questions are provided in Appendix A.

An ineffective survey design can compromise data validity and reliability (Minto et al. 2017). As there are no definitive guidelines for e‐survey design, the survey was tested before dissemination and changes were made to question the design and content based on feedback.

The final survey consisted of 23 questions, some conditional based on prior responses. The number was kept below 30 to avoid reduced response rates and to prevent participants from rushing through the survey, which could lower response quality (Sharma 2022).

The survey was divided into four sections: professional characteristics, general understanding of DCM, knowledge of assessment and management, and areas for further education. It included multiple‐choice, Likert scale questions and free‐text options for additional comments to help substantiate the answers provided.

### 3.2 Participant Eligibility Criteria

The survey was open to UK MSK physiotherapists registered with the Health and Care Professional Council (HCPC) currently treating patients with musculoskeletal conditions.

### 3.3 Participant Recruitment

Recruitment was performed using a combination of purposive, snowball, and convenience sampling via an online invitation containing a secure weblink to the survey via Qualtrics. The survey was open for four weeks from March 2nd, 2024.

To boost responses, the invitation was shared across professional networks and social media, including the interactive Chartered Society of Physiotherapy (iCSP), the Advanced Practice Physiotherapy Network (APPN), the National Spine Network (NSN), and the social media platform X (formerly Twitter). Gatekeeper approval was obtained where necessary.

The invitation link took participants to the participant information leaflet, which could be downloaded for reference. The leaflet detailed the survey's rationale and assured participants of anonymity, with withdrawal only possible before submission. A consent statement was required to access the survey. Participants could review or change responses before submission, with withdrawal possible by not completing or submitting the survey.

Qualtrics software hid participants' IP addresses, ensuring anonymity. Participation was voluntary, with no incentives offered. To optimise response rates, the completion time was judged following testing to be between 10 and 15 minutes (Revilla and Ochoa 2017).

### 3.4 Data Handling

Completed data were stored in a secure, password‐protected folder on YSJ University's OneDrive. It will be kept for 3 years per UK Research Integrity Office guidelines (2021) and YSJ University Ethics Policy (2021).

### 3.5 Data Analysis

Questionnaire data were exported to Excel and reported as descriptive statistics, frequencies, and percentages. All data sets were complete. Text box entries were analysed using thematic analysis involving systematic coding, theme generation, and theme refinement (Braun and Clarke, 2022).

## 4 Results

A total of 108 physiotherapists completed the survey. Table 1 outlines the characteristics of the respondents. Over half (62.96%) had more than 10 years of experience, and 84.6% held senior roles (Agenda for Change Band 7 or higher). The majority worked within the NHS (96.3%) primarily in primary care settings (66%).

**TABLE 1:** Participant characteristics.

	Number of participants	%
Work setting		
NHS	104	96.30
Private (employed)	4	3.70
Private (self‐employed)	0	0.00
Total	108	100.00
NHS employees—Agenda for change banding		
Band 5	2	1.92
Band 6	14	13.46
Band 7	37	35.58
Band 8a	45	43.27
Band 8b	6	5.77
Band 9	0	0.00
Total	104	100.00
NHS employees—Primary or secondary care setting		
Primary care	69	66.35
Secondary care	35	33.65
Total	104	100.00
Years of MSK experience		
1–2 Years	5	4.63
3–4 Years	4	3.70
5–10 Years	31	28.70
11–20 Years	36	33.33
21–30 Years	23	21.30
30+ years	9	8.33
Total	108	100.00
Current work environment/clinic		
Outpatient MSK (generic—NHS & private)	47	43.52
Advanced upper limb	4	3.70
Advanced lower limb	4	3.70
Spinal clinic	19	17.59
FCP	20	18.52
Pain management	1	0.93
Other^a^	13	12.04
Total	108	100.00

^a^ Other data includes Mental Health Inpatients; MSK OPD/FCP; All of the above except pain; 50‐50% Advanced/FCP; General MSK APP service NHS; APP general so spine, UL, LL; Do a mixed role FCP and pain management; ED; FCP and Advanced Practice Clinics/Community Ortho Service Clinics (all areas); Triage interface clinic; Whole body Orthopaedic triage; Fcp one day a week; Musculoskeletal Triage Clinic.

### 4.1 Section 1: General Knowledge and Understanding Among MSK Physiotherapists of DCM

Nearly all respondents (89.81%) reported feeling ‘very’ or ‘fairly’ confident in their understanding of the term ‘degenerative cervical myelopathy’ (DCM). Just over one‐third of participants (39.81%) were aware of the current estimated prevalence of DCM in the adult population, with 42.59% overestimating and 17.59% underestimating it. Slightly under half of the participants (44.4%) indicated they would be more likely to consider a diagnosis of DCM if the patient was over 50 years of age, while 27.78% selected over 60 years. Only 26.85% of respondents correctly identified the current estimated delay to diagnosis as being over 2 years (See Table 2 for full result details).

**TABLE 2:** General knowledge and understanding among MSK physiotherapists of DCM.

	Number of participants	%
How confident are you in your understanding of the term degenerative cervical myelopathy?		
Not at all confident	0	0.00
Slightly confident	11	10.19
Fairly confident	51	47.22
Very confident	46	42.59
Total	108	100.00
How prevalent do you estimate DCM to be in the adult population?		
< 2%	19	17.59
2%–5%	43	39.81
5%–10%	26	24.07
10%–15%	15	13.89
> 15%	5	4.63
Total	108	100.00
From what patient age would you be more likely to consider DCM as a potential diagnosis?		
20+	0	0.00
30+	2	1.85
40+	19	17.59
50+	48	44.44
60+	30	27.78
70+	8	7.41
80+	1	0.93
Total	108	100.00
What is the estimated average time delay in diagnosis for DCM patients from the point of first accessing health care?		
6 weeks	1	0.93
12 weeks	6	5.56
6 months	23	21.30
12 months	19	17.59
2 years	30	27.78
> 2 years	29	26.85
Total	108	100.00

### 4.2 Section 2: Self‐Perceived Confidence in Assessing, Diagnosing, and Managing DCM

#### 4.2.1 DCM Subjective Assessment

Most respondents (86.12%) reported feeling ‘fairly’ or ‘very confident’ in their ability to conduct a comprehensive screening for DCM through subjective questioning. When asked about their understanding of the clinical rationale behind the screening questions, 87.04% expressed being ‘fairly’ or ‘very confident’. Additionally, 86.11% of respondents indicated they were ‘fairly’ or ‘very confident’ in their ability to recognise the signs and symptoms of DCM (See Table 3 for full result details).

**TABLE 3:** Self‐perceived confidence in assessing, diagnosing, and managing DCM.

	Number of participants	%
How confident do you feel in identifying the signs and symptoms of DCM?		
Not at all confident	0	0.00
Slightly confident	15	13.89
Fairly confident	63	58.33
Very confident	30	27.78
Total	108	100.00
How confident are you that you are comprehensively screening for DCM in your subjective questioning?		
Not at all confident	2	1.85
Slightly confident	13	12.04
Fairly confident	65	60.19
Very confident	28	25.93
Total	108	100.00
Are you confident that you fully understand the clinical rationale behind the screening questions you ask?		
Not at all confident	2	1.85
Slightly confident	12	11.11
Fairly confident	61	56.48
Very confident	33	30.56
Total	108	100.00
How confident are you in selecting clinical tests for DCM?		
Not at all confident	3	2.78
Slightly confident	13	12.04
Fairly confident	59	54.63
Very confident	33	30.56
Total	108	100.00
How confident are you at performing clinical testing for DCM?		
Not at all confident	2	1.85
Slightly confident	15	13.89
Fairly confident	59	54.63
Very confident	32	29.63
Total	108	100.00
How confident are you that you fully understand the clinical reasoning behind these clinical tests?		
Not at all confident	4	3.70
Slightly confident	26	24.07
Fairly confident	54	50.00
Very confident	24	22.22
Total	108	100.00
If you suspect DCM from your clinical exam, how confident are you in knowing when imaging is required?		
Not at all confident	2	1.85
Slightly confident	15	13.89
Fairly confident	44	40.74
Very confident	47	43.52
Total	108	100.00
If DCM is confirmed how confident are you in following current guidelines for treatment pathways?		
Not at all confident	7	6.48
Slightly confident	15	13.89
Fairly confident	47	43.52
Very confident	39	36.11
Total	108	100.00
Which of the following investigations is the most important for diagnosing degenerative cervical myelopathy?		
NCS & EMG	0	0.00
MRI CX	103	95.37
CT myelogram	1	0.93
CT C spine	0	0.00
AP & lateral C‐spine radiographs	1	0.93
Dont know	3	2.78
Total	108	96.30
Have your heard of the Japanese Orthopaedic Association (JOA) scale or the modified JOA scale?		
Yes	52	48.15
No	56	51.85
Total	108	100.00
Do you use the Japanese Orthopaedic Association (JOA) scale or the modified JOA scale in your practice?		
Yes	34	65.38
No	18	34.62
Total	52	100.00

#### 4.2.2 Symptom Awareness & Identification

Respondents were asked to identify potential symptoms of degenerative cervical myelopathy (DCM). The most recognised symptoms included reduced grip strength, impaired hand dexterity, clumsiness, and poor balance, followed by paraesthesia and numbness in the upper limbs, falls, paraesthesia and numbness in the lower limbs, weakness and stiffness in the upper limbs, and neck pain. In contrast, the least identified symptoms were abdominal pain, hot flushes or sweating, neck clicking, constipation, tinnitus, and breathing difficulties (See Chart 1a for the results breakdown).

**CHART 1a:**
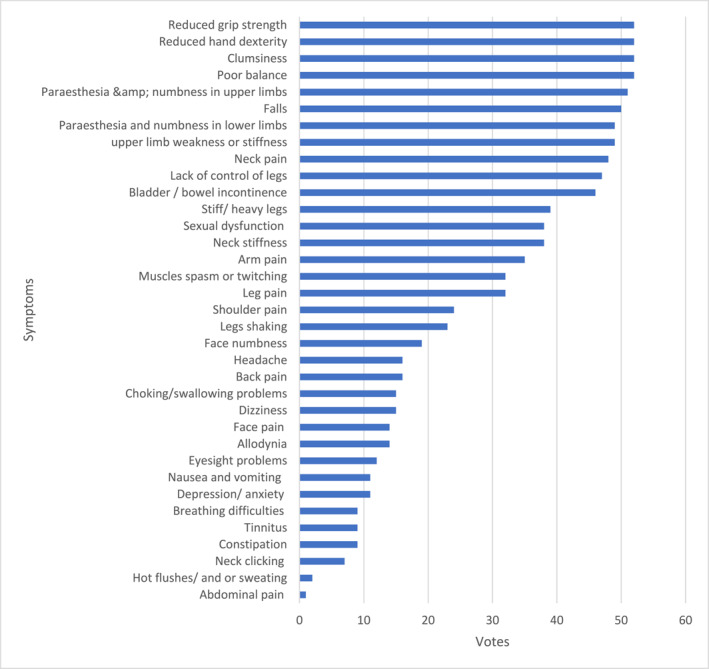
Symptom awareness and identification.

#### 4.2.3 DCM Objective Assessment

Most participants (85.19%) reported being either ‘fairly’ or ‘very confident’ in their ability to select appropriate clinical tests, with 72.22% expressing confidence in the clinical reasoning underlying their choices. Additionally, 84.26% of participants indicated they were ‘fairly’ or ‘very confident’ in their execution of these clinical tests (See Table 3 for full results).

#### 4.2.4 Clinical Test Selection

The most frequently utilised assessments were upper limb motor testing, with 100% of respondents reporting consistent use, followed by upper limb reflexes (98%), Hoffman's reflex (95%), upper limb sensory testing (91%), Babinski sign (87%), lower limb reflexes (86%), lower limb motor testing (84%), Clonus (81%), and lower limb sensory testing (77%). In contrast, Wazir's test was the least known, with 84% of respondents unfamiliar with it, and only 2% reporting occasional use. Similarly, Tromner's test and the finger escape sign were among the least recognised (See Chart 1b).

**CHART 1b:**
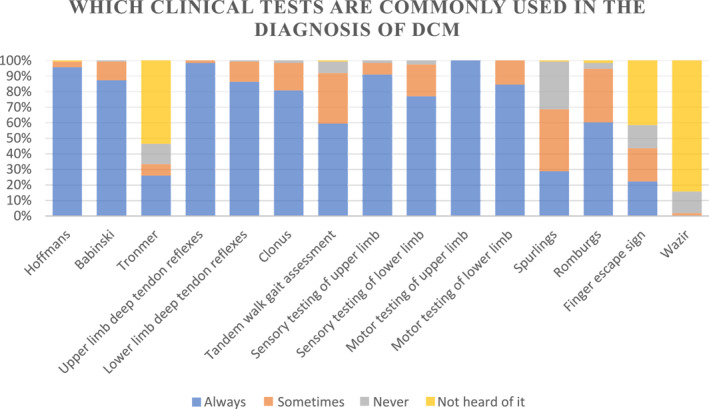
Which clinical tests are most commonly used in the diagnosis of DCM?

#### 4.2.5 Imaging

Most participants (84.26%) reported feeling ‘fairly’ or ‘very’ confident in knowing when imaging was required, with fewer than 2% not confident at all. Most participants (95.37%) identified cervical MRI as their preferred imaging modality for diagnosing DCM, while less than 3% were unsure of which imaging technique to select (See Table 3 for full result details).

#### 4.2.6 Pathways

Most participants (79.63%) felt ‘fairly’ or ‘very’ confident in knowing where and when to refer if a diagnosis of DCM was confirmed, while around 6% reported no confidence at all. Approximately half of the participants were familiar with the modified Japanese Orthopaedic Association (mJOA) score, with two‐thirds of those aware of it using it in their clinics (See Table 3 for full result details).

#### 4.2.7 Fear of Missed Diagnosis

Over half of the participants (58.33%) expressed concern about missing a DCM diagnosis. This group included those who felt they lacked sufficient knowledge about DCM (18.52%) and those who, despite believing they were well‐informed about DCM, still worried about missing a diagnosis (39.81%) (See Table 4 for full result details).

**TABLE 4:** Fear of missed diagnosis.

	Number of participants	%
Do you ever feel anxious or worried about missing DCM as a diagnosis?		
Yes, I feel I know lots but worry I will miss a presentation	43	39.81
Yes, I don't feel I know enough about DCM, and I worry I'll miss a presentation	20	18.52
No, this isn't something I worry or feel anxious about	45	41.67
Total	108	100.00

**FIGURE 1:**
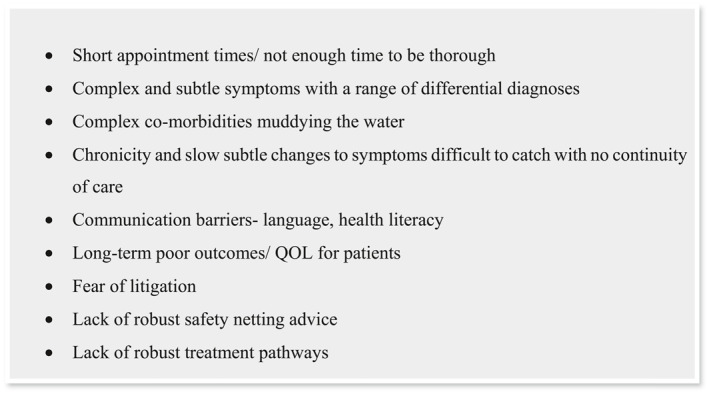
Reasons stated for anxiety and worry.

Reasons stated for anxiety and worry can be found in below: see Figure 1.

### 4.3 Section 3: Self‐Perceived Educational Training Needs of MSK Physiotherapists to Enhance Competence in Assessing, Diagnosing, and Managing DCM

#### 4.3.1 Previous Training on DCM & Future Training Needs

Just under half of the participants (45.37%) believed that the topic of DCM was not covered at all during their undergraduate studies, while 39 participants (36.11%) could not recall. Only four participants (3.7%) felt that the subject had been covered comprehensively (See Table 5 for full result details).

**TABLE 5:** Previous training on DCM & future training needs.

	Number of participants	%
Was DCM covered as part of your undergraduate training?		
Yes, covered thoroughly	4	3.70
Yes, not covered thoroughly	16	14.81
Can't remember	39	36.11
No, not covered	49	45.37
Total	108	100.00
Have you had access to training on DCM as a postgraduate?		
Yes	79	73.15
No	29	26.85
Total	108	100.00
Do you think you would benefit from increased training on DCM?		
Yes	97	89.81
No	11	10.19
Total	108	100.00
Which area do you think you would benefit most from additional training in? (select all that apply)		
Assessment symptomology	54	17.20
Assessment screening questions	59	18.79
Diagnosis clinical tests	76	24.20
Diagnosis imaging	34	10.83
Management (non‐surgical)	57	18.15
Management (surgical)	30	9.55
Other	4	1.27
Total	314	100.00

Most participants (73.15%) had received training on DCM at the postgraduate level. Furthermore, most participants (89.81%) felt they would benefit from additional training on DCM, with diagnostic clinical tests being the most frequently identified area for further training needs.

### 4.4 Section 4: Self‐Perceived Barriers of MSK Physiotherapists to Providing Assessment, Diagnosis, and Management of DCM

The two most frequently identified barriers to the effective assessment, diagnosis, and management of DCM were a lack of guidelines and a lack of training (See Table 6 for full details).

**TABLE 6:** Barriers to providing assessment, diagnosis, and management of DCM.

	Number of participants	%
What do you think are the main barriers to you effectively assessing/diagnosing DCM?		
Lack of knowledge	33	15.71
Lack of confidence	29	13.81
Lack of training	47	22.38
Lack of time	28	13.33
Lack of guidelines	58	27.62
Other	15	7.14
Total	210	100.00

### 4.5 Section 5: Perspectives of MSK Physiotherapists on Improving DCM Care

**FIGURE 2:**
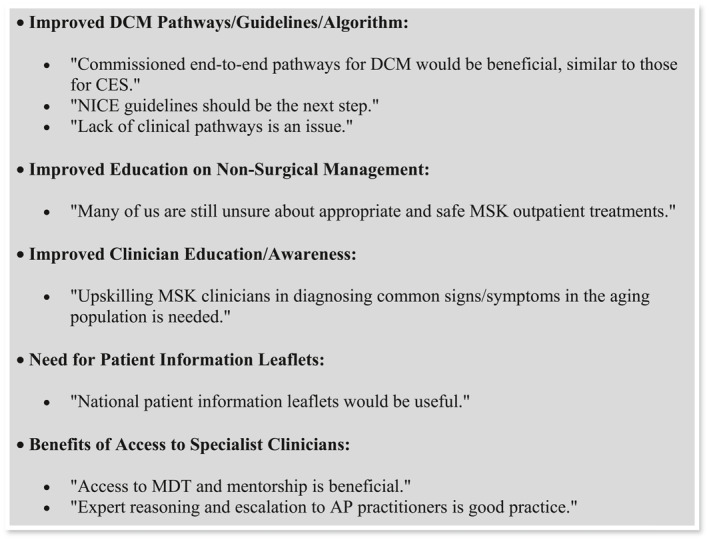
Perspectives of MSK physiotherapists on improving DCM care.

A summary of key points is presented below in Figure 2.

## 5 Discussion

The primary aim of this research was to examine musculoskeletal (MSK) physiotherapists' awareness and knowledge of degenerative cervical myelopathy (DCM). Additionally, the study sought to investigate self‐perceived training needs and perceived barriers to the effective assessment, diagnosis, and management of DCM. The results indicate that MSK physiotherapists report high levels of self‐perceived awareness, knowledge, and confidence in the assessment, diagnosis, and management of DCM. These findings differ from the conclusions of the RECODE DCM consensus project (AO Spine, 2023) and a recent similar survey by Kennedy et al. (2023), both of which concluded that awareness among healthcare professionals is generally low.

One possible explanation for this discrepancy is that previous studies surveyed a broader range of healthcare professionals, including non‐specialist practitioners, such as general practitioners. As this study focused exclusively on MSK clinicians, their specialist knowledge may account for the higher levels of awareness and confidence observed. This is further supported by a deeper analysis of the results, which revealed that those with more years of experience and those working in advanced roles generally scored higher on confidence‐related questions. The recent survey by Kennedy et al. (2023) included physiotherapists alongside general practitioners, chiropractors, osteopaths, and nurse practitioners. While the majority of participants were physiotherapists, it is unclear whether they were MSK specialists, which may explain the lower levels of knowledge and awareness reported in that study. Additionally, as Kennedy et al.’s survey was conducted in New Zealand, the results may not be generalisable to the UK, where differences in healthcare education and systems may contribute to variations in awareness.

Another potential explanation for the high levels of knowledge and awareness observed in this study could be the recent efforts by the RECODE DCM team to raise awareness. Since the initiation of this research, a DCM educational video has been disseminated via social media as part of the ‘Raising Awareness of Degenerative CErvical Myelopathy Amongst Physiotherapists’ (RADCEM‐PHYSIO) campaign (Tabrah 2024). Supporting this hypothesis, most participants reported having received post‐graduate training on DCM, though it was not specifically linked to the aforementioned campaign.

It is acknowledged that certain limitations in the study design may have influenced the results, potentially giving the impression of greater awareness and knowledge due to bias. Physiotherapists with specific interests in DCM may have been more inclined to respond to the survey. This effect could have been amplified by the survey's promotion of advanced and spinal‐specific networks, in addition to non‐specialist musculoskeletal (MSK) platforms. This may explain the higher participation of experienced clinicians working in advanced practice and specialist spinal clinics. These factors increase the risk of non‐response and self‐selection bias, limiting the generalisability of the findings to less experienced MSK clinicians.

It remains unclear why fewer responses were received from less experienced clinicians. One hypothesis is that a lack of confidence in the subject may have deterred them from participating, possibly due to concerns about appearing uninformed, despite assurances of anonymity. Alternatively, it could suggest that DCM is not a prominent consideration for less experienced clinicians, a notion supported by the survey's finding that 88% of participants either believed DCM was not covered at the undergraduate level or could not recall it being included. Additionally, the online nature of the survey, with no control over respondent behaviour, raises the possibility of social desirability bias, where participants may have sought external information before answering the questions, thereby distorting the results.

Despite the potential biases in this survey, the findings still raise an important question: if awareness of DCM is high, why does evidence suggest such significant rates of missed and delayed diagnoses (Davies et al. 2018)? This issue has been explored by Davies, Mowforth, et al. (2022), who examined the evidence suggesting low awareness of DCM. They concluded that high theoretical knowledge does not necessarily translate to a high index of clinical suspicion in practice. One key factor appears to be the complex and varied symptomatology of DCM, a point reinforced by the findings of this survey. Although participants rated themselves as confident in their assessment skills, concerns about missed diagnoses were frequently linked to the complexity of DCM symptoms.

Two studies have specifically reviewed the intricacy of DCM symptom presentation. Munro et al. (2023) compiled a list of conventional and non‐conventional symptoms, while Jiang et al. (2024) categorised symptoms into the most and least common. The results of this survey indicate that MSK physiotherapists are highly aware of the common and conventional symptoms identified by Jiang et al. (2024) and Munro et al. (2023). However, while Jiang et al.'s (2024) scoping review offers a comprehensive examination of DCM symptoms, its conclusions are limited by the lack of studies featuring control groups, which restricts the ability to determine symptom specificity. This lack of specificity likely contributes to the high rates of missed diagnoses. For example, neck pain was identified as the most frequently reported area of pain (Jiang et al. 2024), but neck pain is not unique to DCM, as it is one of the most common MSK complaints (Kazeminasab et al. 2022). The most frequent and sensitive symptoms associated with DCM were generalised paraesthesia, hand numbness, and hand paraesthesia (Jiang et al. 2024). Although these symptoms were among the most recognised by the participants in this survey, indicating good awareness, they are also present in other conditions, such as cervical radiculopathy, peripheral neuropathy, and vitamin D deficiency, which further complicates the diagnostic process.

The survey results suggest lower awareness of unconventional symptoms. However, it is difficult to ascertain how much this contributes to diagnostic delays as research evaluating the diagnostic value of these symptoms is limited. One study proposes that unconventional symptoms, such as chest pain, may help differentiate DCM from other conditions (Kobayashi et al. 2010), indicating that education on these less typical symptoms could enhance early detection. Nevertheless, this study was based on a small sample of 68 patients, all of whom had confirmed DCM diagnoses, limiting the generalisability and validity of its conclusions. Further high‐quality research is needed to establish the diagnostic importance and significance of these unconventional symptoms. Overall, despite good awareness of common symptoms, diagnosis remains challenging due to the complexity and variability of symptom presentations. Additional high‐quality research is required to clarify the prevalence of DCM symptoms and guide clinicians on which symptoms are most indicative of the condition.

The survey results suggest that MSK physiotherapists feel confident in selecting, performing, and understanding clinical tests for DCM. This finding contrasts with the notion that clinicians often lack confidence in conducting neurological assessments, a phenomenon referred to as ‘neurophobia’ (Nicholl and Appleton 2015; Javaid et al. 2018). The high level of experience among participants may help explain this disparity; however, it is important to recognise that self‐assessment of skills can be inaccurate (Bryan and Lindsay 2017). Thus, these results should be interpreted with caution. Confidence is also considered a fluid concept, influenced by various factors, including stress and uncertainty (Ilgen et al. 2019). The survey highlighted anxiety surrounding missed diagnoses, with approximately 50% of participants expressing concern about overlooking a diagnosis due to various factors. Consequently, real‐time clinical confidence may differ from theoretical self‐assessments of confidence.

In addition to self‐rated confidence, this survey examined the clinical tests that participants utilised most frequently. A systematic review by Jiang et al. (2023) identified the most sensitive clinical tests for diagnosing DCM as the Tromner sign and hyperreflexia, while the most specific tests included Babinski, Tromner, clonus, and the inverted supinator sign. The findings of this survey indicate a generally good correlation between the tests used most frequently and those reported to have the highest sensitivity and specificity. However, there was one notable exception: while nearly all participants indicated they ‘always’ used upper and lower limb reflex testing and most consistently employed the Babinski and Clonus tests, the Tromner test was among the least known and least utilised. Despite being one of the few tests with both high sensitivity and specificity (Jiang et al. 2023), the Tromner test was not widely adopted. In contrast, the Hoffman test was reported as ‘always used’ by 95% of participants, despite its lower sensitivity and specificity compared to the Tromner test. This highlights the need for increased awareness of the Tromner test among clinicians.

It is also important to highlight that no correlation has been established between positive clinical tests and disease severity (Jiang et al. 2023), with up to 20% of patients with DCM not displaying any of these examination findings (J. M. Rhee et al. 2009). This may help elucidate why diagnoses are frequently overlooked, despite clinicians' overall awareness and knowledge. Further research is needed to provide clinicians with improved guidance on which combinations of tests produce the most valid and reliable outcomes.

As previously highlighted, despite generally high levels of confidence and awareness regarding DCM, there remains considerable concern and anxiety about missing a diagnosis, which is often linked to the complexity of DCM presentations and the potential consequences of a missed diagnosis for both the patient and the clinician. These apprehensions mirror those expressed by clinicians when discussing anxiety related to the management of other serious spinal pathologies, such as cauda equina syndrome (CES) (Paling and Hebron 2021; Yeowell et al. 2023; Paling and Hebron 2021 identified several strategies that physiotherapists found helpful in alleviating anxiety when dealing with serious pathologies. These strategies included the importance of teamwork and shared responsibility, collaboration with experienced clinicians, and the utility of clear management pathways. Such findings resonate with the results of this survey. Conducting further qualitative research to gain deeper insights into the anxieties surrounding DCM could provide valuable information and potential solutions to address the concerns felt by physiotherapists.

In line with the aforementioned points, despite survey results indicating that most participants were confident in their knowledge of when and where to refer patients with DCM, a significant barrier to effective DCM care identified was the lack of diagnostic and treatment guidelines, along with a desire for increased training on non‐surgical management options.

Currently, MRI is the gold standard for diagnosing DCM. The findings of this survey suggest that most participants are aware of this fact; however, data regarding how many participants have the authority to request MRI scans were not collected. This information would have been valuable in assessing whether limited access to imaging poses a barrier to accurate diagnosis. It is hypothesised that most MSK physiotherapists in non‐specialist roles lack access to imaging requests, which may explain their strong interest in a diagnostic algorithm to aid in their assessments. Although international guidelines for the management of DCM exist (Fehlings et al. 2017), the results of this survey suggest that they are either poorly understood or deemed unhelpful by MSK physiotherapists. There is a specific desire for guidance on non‐surgical management strategies.

Most research on DCM interventions and subsequent guidelines focus on surgical treatment options, with limited investigation into non‐surgical interventions. The international guidelines recommend ‘a supervised trial of structured rehabilitation for patients with mild DCM’ (Fehlings et al. 2017), but the authors acknowledge that these recommendations are based on weak evidence, which limits their applicability. Furthermore, there are few guidelines detailing what non‐surgical interventions or ‘structured rehabilitation’ should entail. One systematic review concluded that there is insufficient evidence to support the recommendation of non‐surgical management options (Tetreault et al. 2017). Another systematic review found that non‐operative treatment for patients with mild DCM yields outcomes similar to surgical options. However, this conclusion was based on low‐level evidence, and rates of hospitalisation for subsequent spinal cord injury were significantly higher in patients receiving initial conservative treatment compared with those managed operatively (J. Rhee et al. 2017). A survey of DCM patients indicated that few benefited from physiotherapy and that provision was inconsistent (Butler et al. 2022). Brannigan et al. (2024) examined management strategies for mild DCM symptoms among healthcare professionals to quantify variability and concluded that no consensus exists. However, these findings may be influenced by selection bias as the majority of the 699 participants were surgeons. In conclusion, there is a pressing need for high‐quality research exploring non‐surgical management options, which aligns with the findings of this survey.

The modified Japanese Orthopaedic Association Scale (mJOA) is a validated tool for assessing the severity of DCM (Kopjar et al. 2015) and is also instrumental in determining when a surgical referral is necessary (Tetreault et al. 2017). The findings from this survey indicate a 50/50 split in awareness of the mJOA, underscoring the need for heightened awareness of this tool. Limited familiarity with the mJOA may contribute to delayed referrals for surgical intervention, potentially leading to poorer long‐term outcomes (Behrbalk et al. 2013). While this survey focused solely on awareness of the mJOA, other assessment tools are available. The National Institutes of Health Toolbox has been reported to be sensitive for detecting DCM (Muhammad et al. 2023), though the small sample size of this study diminishes the reliability and validity of its conclusions. Another tool, the Nurick grading system, has been reported to be less sensitive than the mJOA (Tetreault et al. 2013). Future research comparing these tools to guide clinicians in selecting the most sensitive assessments would be advantageous, especially given the push for increased awareness and earlier diagnosis. Such advancements could lead to a greater emphasis on non‐surgical management options and improved methods for monitoring symptoms.

### Barriers and Learning Needs:

- *The most identified barrier to managing DCM was insufficient training*.
- *The most frequently identified training need was clinical testing*. This is intriguing considering the self‐reported high confidence in this area. However, this can likely be attributed to the broad array of clinical tests available and the limited consensus guidance as previously discussed.
- *Training in non‐surgical management, symptomology and screening questions were also identified as training needs*. This is not unexpected, considering the limited robust research and guidelines available to clinicians, as previously discussed.
- *A lack of training at the undergraduate level was also highlighted by this survey*. This aligns with the findings of other studies (Brannigan et al. 2022; Waqar et al. 2020). The question of whether resources would be better allocated to enhancing training at the undergraduate level or whether the emphasis for DCM education should be placed on postgraduate training is beyond the scope of this survey but warrants further discussion.

## 6 Strengths and Limitations

To the best of the author's knowledge, this is the first survey to investigate the knowledge and understanding of DCM among MSK physiotherapists. The findings provide valuable insights into contemporary physiotherapy practice. However, as the study exclusively included UK physiotherapists, the results are confined to this context, and broader sampling would enhance the data's international relevance.

A significant limitation is the high level of experience among participants, which may skew the results and affect their generalisability. Additionally, the majority of respondents were employed by the NHS, which may limit the applicability of the findings to private or self‐employed physiotherapists. The reasons for this discrepancy are unclear, as there were no restrictions on the survey platforms used. Furthermore, not all MSK physiotherapists may have access to online resources, leaving some responses unknown. Self‐selection bias could also influence the reliability of the data; although this is a concern, it is believed that participants were unlikely to misrepresent their qualifications.

High response rates help mitigate selection bias and enhance precision and validity (Burns et al. 2008). However, determining the response rate proved challenging due to the lack of clear statistics on the number of UK MSK physiotherapists. In a recent similar survey, Kennedy et al. (2023) reported responses from 255 clinicians, surveying a range of primary care professionals rather than focussing solely on physiotherapists, thus suggesting a higher expected response rate. While the sample size of 108 is relatively small, it is comparable to similar studies (Coulthard et al. 2021; Rath et al. 2021) and is considered acceptable.

## 7 Conclusion

Previous studies have indicated a low level of awareness regarding DCM among healthcare professionals. In contrast, this survey focussing on MSK physiotherapists reveals a high level of awareness and confidence in the assessment and management of DCM, underscoring the importance of leveraging the expertise of MSK physiotherapists in the global initiative to enhance DCM care.

Despite this generally high awareness, there is evidence of clinician anxiety surrounding the condition, along with a clear demand for additional training. The most frequently cited barrier to effective management was a lack of training, particularly in clinical testing, symptomatology, and non‐surgical management. Participants expressed a strong interest in the development of a DCM algorithm to assist with its assessment, diagnosis, and management.

Many participants reported feeling inadequately trained at the undergraduate level. Engaging with higher education providers could improve awareness of DCM from the outset of a clinician's career, thereby embedding it into their professional mindset early on. Further dialogue between DCM experts and higher education institutions on this issue could be highly beneficial.

Addressing these findings could lead to significant improvements in the quality of care, ultimately enhancing the long‐term outcomes and quality of life for individuals suffering from DCM. Additional benefits could include a reduction in clinician anxiety and considerable cost savings for society.

## Author Contributions

Study concept and design: Esther Dawson. Acquisition of data: Esther Dawson. Analysis and interpretation of data: Esther Dawson. Draughting of the manuscript: Esther Dawson and Fi Macintosh. Critical revision of the manuscript: Fi Macintosh. Study supervision: Fi Macintosh.

## Ethics Statement

The Ethics Committee of the University of York St John granted ethical approval. Reference: PHC7022M/CB/05022024/ED/FM.

## Conflicts of Interest

The authors declare no conflicts of interest.

## Data Availability

The data that support the findings of this study are available from the corresponding author upon reasonable request.

## Appendix A Survey Questions

Q1 Do you work in an NHS or private healthcare setting?

Q2 What agenda for changing the banding level are you currently employed at?

Q3 Do you work in a Primary or Secondary care setting?

Q4 How many years have you worked within MSK physiotherapy?

Q5 What area best describes the MSK area in which you currently work?

Q6 Was DCM covered as part of your undergraduate training?

Q7 Have you had access to training on DCM as a postgraduate?

This next set of questions explores your knowledge and awareness of DCM.

Q8 How confident are you in your understanding of the term degenerative cervical myelopathy?

Q9 How prevalent do you estimate DCM to be in the adult population?

Q10 From what patient age would you be more likely to consider DCM as a potential diagnosis?

Q11 What is the estimated average time delay in diagnosis for DCM patients from the point of first accessing health care?

The following questions will ask you to state how confident you feel about different aspects of assessing/diagnosing/managing DCM….

Q12 How confident do you feel in identifying the signs and symptoms of DCM?

Q13 How confident are you that you are comprehensively screening for DCM in your subjective questioning?

Q14 Are you confident that you fully understand the clinical rationale behind the screening questions you ask?

Q15 How confident are you in selecting clinical tests for DCM?

Q16 How confident are you at performing clinical testing for DCM?

Q17 How confident are you that you fully understand the clinical reasoning behind these clinical tests?

Q18 If you suspect DCM from your clinical exam, how confident are you in knowing when imaging is required?

Q19 If DCM is confirmed, how confident are you in following current guidelines for treatment pathways? for example knowing where and how urgently to refer?

Q20 Do you ever feel anxious or worried about missing DCM as a diagnosis?

Q21 Why do you feel anxious or worried? Please use the text box to briefly explain your reasons.

These next questions will explore your knowledge of DCM symptoms, assessment and management.

Q22 What symptoms would you identify as potential symptoms of DCM (select all that apply)?

Poor balance (1) Lack of control of legs (2) Stiff/heavy legs (3) Falls (4) Clumsiness (4) Reduced hand dexterity (6) Reduced grip strength (7) upper limb weakness or stiffness (8) paraesthesia & numbness in upper limbs (9) paraesthesia and numbness in lower limbs (10) Neck pain (11) Shoulder pain (12) Back pain (13) Arm pain (14) Leg pain (15) Neck stiffness (16) Neck clicking (17) Allodynia (18) Bladder/bowel incontinence (19) Constipation (20) Hot flushes/and or sweating (21) Abdominal pain (22) Face pain (23) Face numbness (24) Depression/anxiety (25) Legs shaking (26) Muscles spasm or twitching (27) Dizziness (28) Nausea and vomiting (29) Choking/swallowing problems (30)Headache (31)Tinnitus (32) Eyesight problems (33) Sexual dysfunction (34) Breathing difficulties (35).

Q23 Which clinical tests do you use as part of your assessment if suspecting DCM (select all that apply)?

Q24 Which of the following investigations is the most important for diagnosing degenerative cervical myelopathy?

Q25 Have you heard of the Japanese Orthopaedic Association (JOA) scale or the modified JOA Scale?

Q26 Do you use the Japanese Orthopaedic Association (JOA) scale or the modified JOA scale in your practice?

Q27 What do you think are the main barriers to effectively assessing/diagnosing DCM? Select all that apply.

Q28 Do you think you would benefit from increased training on DCM?

Q29 Which area do you think you would benefit most from additional training in? Select all that apply.

Q30 Do you have any other thoughts surrounding DCM assessment/diagnosis/management that you would like to add?
